# Comparison of *PLZF* Gene Expression between Pluripotent Stem Cells and Testicular Germ Cells

**DOI:** 10.22074/cellj.2020.6532

**Published:** 2019-09-08

**Authors:** Hossein Azizi, Morteza Koruji, Thomas Skutella

**Affiliations:** 1 Faculty of Biotechnology, Amol University of Special Modern Technologies, Amol, Iran; 2 Cellular and Molecular Research Center and Department of Anatomical Sciences, Iran University of Medical Sciences (IUMS), Tehran, Iran; 3 Institute for Anatomy and Cell Biology, Medical Faculty, University of Heidelberg, Im Neuenheimer Feld 307, Heidelberg, Germany

**Keywords:** Embryonic Stem Cells, Germ Cells, *PLZF* Gene, Spermatogonial Stem Cells

## Abstract

**Objective:**

Spermatogonial stem cells (SSCs), as unipotent stem cells, are responsible for the production of sperm throughout the male’s life. Zinc finger and BTB domain containing 16 (*ZBTB16/PLZF*) genes provide various functions in the cell development, signaling pathway, growth regulatory and differentiation. Here, we aimed to investigate expression of the PLZF germ cell gene marker in testis, SSCs, pluripotent embryonic stem cells (ES cells) and ES-like cells of mouse testis.

**Materials and Methods:**

In this experimental study, we examined the expression of the PLZF germ cell marker in the testis section and testicular cell culture of neonate and adult mice by immunohistochemistry (IMH), immunocytochemistry (ICC) and Fluidigm Real-Time polymerase chain reaction (PCR).

**Results:**

IMH data indicated that the PLZF protein was localized in the neonate testis cells of the tubules center as well as the basal compartment of adult testis seminiferous tubules. Counting PLZF IMH-positive cells in the sections of seminiferous tubules of adult and neonate testis revealed significant expression of positive cells in adult testis compared to the neonate (P<0.05). Under *in vitro* conditions, isolated SSC colonies were strongly ICC-positive for the PLZF germ cell marker, while ES cells and ES-like cells were negative for PLZF. Fluidigm Real-Time-PCR analysis demonstrated a significant expression of the *PLZF* germ cell gene in the neonate and adult SSCs, compared to ES cells and ES-like cells (P<0.05).

**Conclusion:**

These results indicate that PLZF is a specific transcription factor of testicular germ cell proliferation, but it is down- regulated in pluripotent germ cells. This can be supportive for the analysis of germ cells development both *in vitro* and *in vivo*.

## Introduction

Germ cells are formed and matured during early embryogenesis from primordial germ cells (PGCs) (1). Spermatogonial stem cells (SSCs) are the adult stem cells located in the basal membrane of seminiferous tubules of testis. They receive cytokines from somatic cells including Sertoli cells, blood vessels, Leydig cells and macrophages. SSCs can be isolated by fluorescence-activated cell sorting (FACS), magnetic-activated cell sorting (MACS), matrix selection and morphology-based selection (2-4). SSCs have the potential for conversion into embryonic stem (ES)-like pluripotent stem cells under defined *in vitro* culture conditions (2-5). 

Extrinsic secreted growth factors from the SSCs niche and intrinsic gene expression play a crucial role in the maintenance of SSCs (2, 6). Extrinsic factors which are produced and secreted by Sertoli cells include glial cell- derived neurotrophic factor (GDNF) and KIT ligand (KITL) (7). Intrinsic factors include PLZF (8, 9), ETV5 (10), Taf4b (11), Bcl6b (12), Pou5f1, Nrg1, Nanog and Gja1 (13-15) as well as Gfra1 and RET (16). The transcription factor PLZF, as a transcriptional repressor that regulates the epigenetic state of undifferentiated cells, is involved in different cellular functions such as cell proliferation, apoptosis and differentiation during spermatogenesis, neurogenesis and embryonic development (8, 17, 18). 

Filipponi et al. (19) demonstrated that PLZF directly represses the transcription of kit, a marker of spermatogonial differentiation. PLZF plays an essential role in the self- renewal and maintenance of the SSC in the testis niche (8). It has been shown that PLZF is co-expressed with Oct4 in undifferentiated spermatogonia. It has also been demonstratedthat loss of the encoding *PLZF* gene produces limited numbers of normal spermatozoa and then leading progressively to the lack of respected germline after birth. During embryogenesis, PLZF regulates the stage of gene expressions of limb and axial skeletal patterning (8, 9, 20). During limb development, it has been demonstrated that PLZF has genetic relationship with *Gli3* and *Hox5* genes (21, 22). Previous studies showed that PLZF was expressed in testis and SSCs, therefore recognized as a SSC marker (23-25). In the present research we have extended our study to the expression of PLZF marker in the neonate and adult testis sections, isolated SSCs, ES cells and generated ES-like cells from mouse testicular culture to evaluate if PLZF has the same expression pattern in bothtesticular germ cells and pluripotent stem cells. The resultsindicated that PLZF is clearly expressed in germ cells, but not in pluripotent stem cells.

## Materials and Methods

### Digestion and culture of testicular cells

In this experimental study, neonate and adult C57BL/6 mouse strain testis cells were isolated by collagenase IV (0.5 mg/ml), DNase (0.5 mg/ml) and Dispase (0.5 mg/ml, all from Sigma-Aldrich, USA) enzymatic digestion solution solved in Hank’s Balanced Salt Solution (HBSS) buffer containing Ca^2+^ and Mg^2+^ (PAA, USA). Digested testicular cells was cultured in SSC condition medium, composed of StemPro-34 medium, 6 mg/ml D+glucose (Sigma-Aldrich, USA), 1% L-glutamine (PAA, USA), 1% N2-supplement (Invitrogen, USA), 0.1% ß-mercaptoethanol (Invitrogen, USA), 1% penicillin/streptomycin (Pen/Strep, PAA, USA), 5 µg/ml bovine serum albumin (BSA, Sigma-Aldrich, USA), 1% non-essential amino acids (NEAA, PAA, USA), 30 ng/ ml estradiol (Sigma-Aldrich, USA), 60 ng/ml progesterone (Sigma-Aldrich, USA), 20 ng/ml epidermal growth factor (EGF, Sigma-Aldrich, USA), 10 ng/ml fibroblast growth factor (FGF, Sigma-Aldrich, USA), 8 ng/ml GDNF (Sigma- Aldrich, USA), 100 U/ml human leukemia inhibitory factor (LIF, Millipore, USA), 1% Minimum Essential Medium (MEM) vitamins (PAA, USA), 1% ES cell qualified fetal bovine serum (FBS, Gibco, USA), 100 µg/ml ascorbic acid, 30 µg/ml pyruvic acid and 1 µl/ml DL-lactic acid (all from Sigma Aldrich, USA) at 37°C and 5% CO_2_ in air (2). 

### Culture of the embryonic stem and ES-like cells

ES and ES-like cell lines were originated from our previous study (2). These cells were cultured in medium with KO-DMEM, composed of 1% NEAA solution, 15% FBS, 1% L-glutamine, 0.1% ß-mercaptoethanol, LIF at a final concentration of 1000 U/ml and 1% Pen/Strep (2). 

### Gene expression analyses on the Fluidigm Biomark system 

Quantity of the *PLZF* gene expression (Mm01176868_ m1) in the neonate SSCs, adult SSCs, ES cells, and ES-like cells were examined by dynamic array chips(Fluidigm). Glyceraldehyde-3-phosphate dehydrogenase (*GAPDH*, Mm99999915_g1) was used as housekeepinggene for normalization. Cultured cells were selected with amicromanipulator, lysed with lysis buffer solution containing 1.3 µl TE buffer, 0.2 µl RT/Taq Superscript III (Invitrogen, USA), 9 µl RT-PreAmp Master Mix, 5.0 µl Cells Direct2× Reaction Mix (Invitrogen, USA), and 2.5 µl 0.2× assay pool. Using TaqMan real-time PCR on the BioMark Real- Time quantitative PCR (qPCR) system, the amount of RNA- targeted copies was evaluated. Samples were examined intwo technical repeats. The Ct values were analyzed by GenEx software from the MultiD analysis (2, 3, 6). 

### Immunocytochemical staining

SSCs, ES cells and ES-like cells were fixed with 4% paraformaldehyde and then permeabilized with 0.1% Triton/PBS. Cells were blocked with 1% BSA/PBS and followed by incubation with primary antibody PLZF. In the next step, we used overnight incubation fluorochrome species-specific secondary antibody and the labeled cells were nuclear counterstained with 0.2 µg/ml of 4’, 6-diamidino-2-phenylindole (DAPI) dye. The labeled positive cells were studied with a confocal microscope Zeiss LSM 700 (Germany), and images were acquired using a Zeiss LSM-TPMT camera (Germany) (2, 26-28). 

### Tissue processing for immunohistofluorescence staining 

Mouse testis tissue was washed with PBS and fixed in 4% paraformaldehyde. Dehydrated tissue was surrounded in Paraplast Plus and cut with a microtome machine at 10 µmthickness. Testis tissue sections were mounted on SuperfrostPlus slides and kept at room temperature until used. Forprocessing of immunohistofluorescence staining, sampleswere washed with xylene followed by gradually replacingwith water in ethanol before staining. For the tissue sections, antigen retrieval was performed by heat-induced epitoperetrieval at 95°C for 20 minutes, non-specific binding site oftissue samples was blocked with 10% serum/0.3% Triton inPBS. The experiment of immunofluorescence staining for these samples was continued as explained above (2).

### Statistical analysis

The expression of PLZF in the indicated groups was calculated using one-way analysis of variance (ANOVA), continued with the Tukey’s post-hoc tests (t Test) and compared with the non-parametric Mann-Whitney’s test. The difference among groups was considered statistically if P<0.05. 

## Results

**Fig.1:**
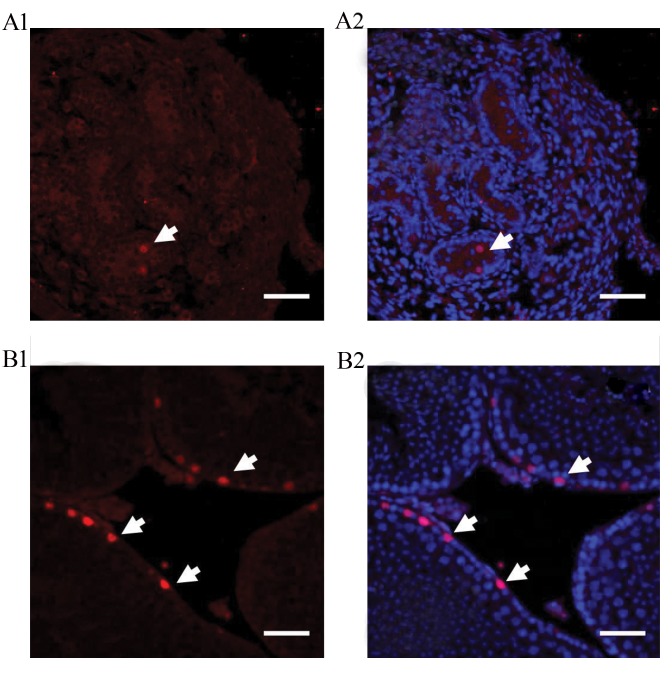
Immunohistochemistry characterization of PLZF in testis section. A1. PLZF expression in neonate, A2. Representation of the merged images with DAPI, B1. PLZF expression in Adult, and B2. Representation of the merged images with DAPI. PLZF; Red and DAPI; Blue.

**Fig.2:**
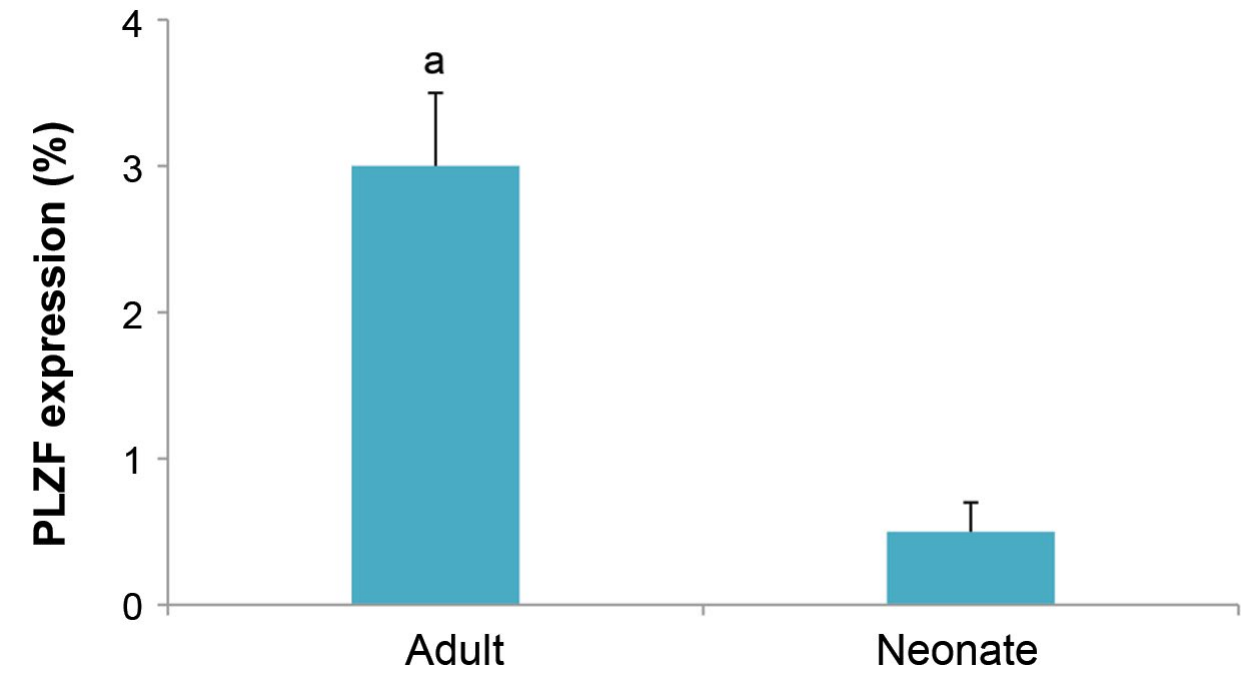
PLZF positive cell counting in testis section. Counting PLZF positive cells in the sections of neonate and adult testes. Number of PLZF positive cells in the adult testis was higher than neonate. a; At least P<0.05 versus other groups. Data are presented as mean ± SD.

**Fig.3:**
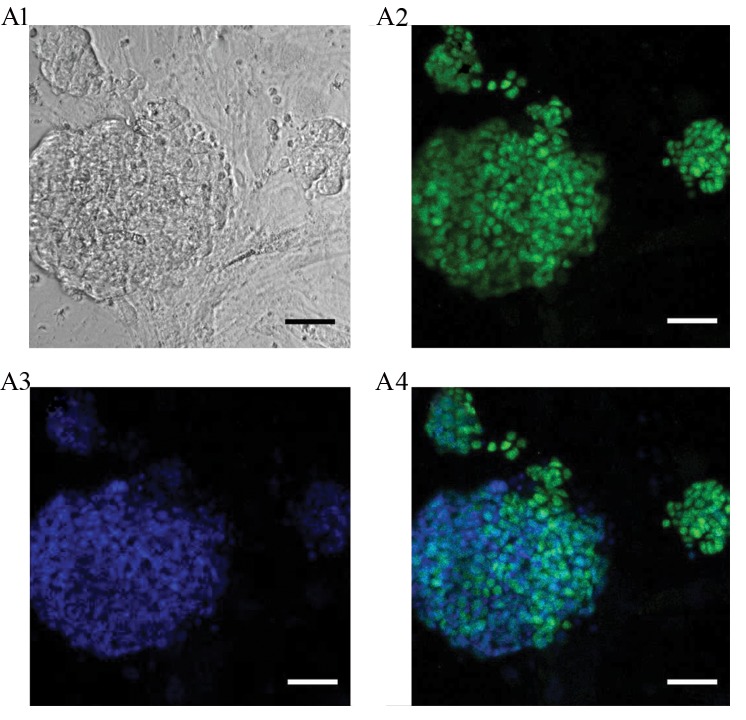
Immunocytochemical characterization of PLZF in spermatogonial stem cells (SSCs). Immunocytochemistry analysis of PLZF expression in the SSC (scale bar: 50 µm). **A1.** Bright field, **A2.** Green fluorescence shows PLZF expression, **A3. **Blue shows DAPI, and **A4.** Representation of the merged images.

**Fig.4:**
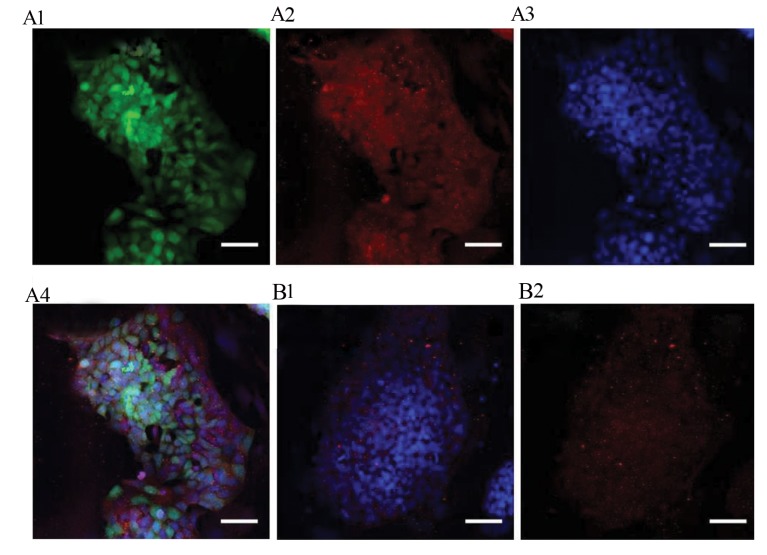
Immunocytochemical characterization of PLZF in the pluripotent cells. Immunocytochemistry analysis showed negative expression of PLZF in the embryonic stem (ES)-like and ES cells (scale bar: 50 µm). **A1. **ES-like, green fluorescence for Oct4, **A2.** ES-like, red fluorescence for PLZF, **A3.** ES-like, blue fluorescence for DAPI, **A4. **ES-like, merged images, **B1.** ES, blue fluorescence for DAPI, and** B2.** ES, red fluorescence fluorescence for PLZF.

**Fig.5:**
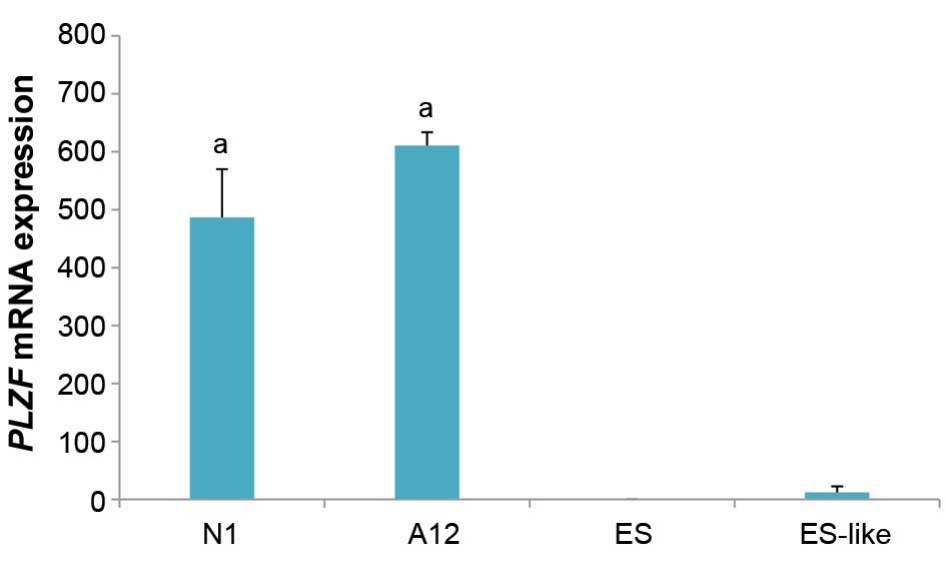
mRNA expression of *PLZF* gene. Fluidigm quantitative polymerase chain reaction (PCR) analysis for *PLZF* expression in the neonate (N1), 12-weeks testis (A12), ES-like and ES cells (a; at least P<0.05 versus other groups). Significant *PLZF* expression levels difference in neonate and adult SSCs compared to ES-like and ES cells. Data are presented as mean ± SD.

We first studied the localization of PLZF in the neonate and adult mouse testis (Fig .1). Immunohistochemical analysis for the cross-section of testis demonstratedthat PLZF protein was expressed in the cells located on the basal membrane of adult testis seminiferous tubule, while in the neonate testis, these cells were located in the center of the tubules (Fig .1). Counting PLZF positive cells in the testis sections of the adult and neonate testis revealed significantly higher expression (P<0.05) of these cells in the adult compared to neonate (Fig .2). Furthermore, neonate and adult SSCs, ES cells and ES- like cells were cultivated *in vitro*, in the defined medium to investigate *PLZF* expression. Neonate and adult SSCs were isolated after enzyme digestion and generated cells cultivated in the presence of growth factors supporting SSC cultivation (Fig .3). Characterization of the isolated SSCs was conducted as described in our former study (2). Immunocytochemistry (ICC) analysis revealed that SSCs were positive, while pluripotent ES and ES-like cells were negative for the PLZF protein (Figes.3, 4). ES-like cell lines containing promoter-reporter *Oct4-GFP* transgenicmice revealed that these pluripotent cells were positive for Oct-4, but they were negative for PLZF (Fig .4). Similarly, Fluidigm real-time RT-PCR results showed significant *PLZF* gene expression in the neonate and 12-weeks old SSCs, compared to ES cells and ES-like cells (P<0.05, Fig .5). 

## Discussion

It has been demonstrated that PLZF transcription factor is a key regulator in SSCs (2). Our histological analysis specified localization of the PLZF positive cells in the center of neonatal testicular cords and basal compartment of the seminiferous tubules of adult testis, co-localized with Oct4 positive cells. PLZF/Oct4 co-localization, in a few single SSCs attached to the basal membrane, implies that these cells are SSCs, but not progenitor cells. The cultured SSCs, which are grown under GDNF stimulation, are also positive for PLZF. Although the number of PLZF positive cells in adult testis was higher than neonate, PLZF mRNA expression level in the neonate and adult SSCs was similar. Protein analyses using immunohisto/ cytochemistry revealed that PLZF was expressed in SSC, but neither in the differentiating germ cells nor in the ES-like cells directly generated from SSCs. It can be concluded that PLZF is down-regulated during both differentiation (spermatogenesis) and conversion of the unipotent SSCs into pluripotent ES-like cells. Similarly, pluripotent ES cells generated from the inner cell mass were negative for PLZF. This finding was also confirmed by Fluidigm real-time RT-PCR and ICC. These observations imply that PLZF strictly bind to and hold the molecular state of a stem cell SCCs. It is proposed that PLZF is a transcriptional repressor and activator involved in the control of SCC (29).

In undifferentiated spermatogonia, it has been shown that PLZF is co-expressed with Oct4. Mutations in the PLZF gene restrict the numbers of spermatozoa cells (9). Mutations in the PLZF display a progressive defect of SSCs and structure of the seminiferous tubule, while the function of supporting Sertoli cells is normal (20). In type A and B spermatogonia, PLZF was found to be localized in the nucleus of undifferentiated SSCs of zebrafish (30). Further studies in SSCs have indicated that the PLZF mutant shows an increase of c-Kit expression (as a marker required for differentiated SSCs), implying that PLZF maintains pool of the SSCs (19). It has been demonstrated that PLZF suppresses transcription activity of the retinoic acid receptors (31). 

Although PLZF expression is positive in undifferentiated cells of stem cell compartment near the basement membrane of adult mouse testis seminiferous tubules but not in spermatocytes, it is unknown whether or not PLZF expression is necessary for initiating differentiation of the SSCs towards spermatocytes. It has been well documented that PLZF plays an important role in the self-renewal and maintenance of gonocytes and undifferentiated spermatogonia (8). PLZF has been demonstrated as a distinguished marker for the isolation of human (23, 32, 33), mouse (24, 34) and sheep SSCs in testicular culture (35). 

It is well-known that PLZF can function as both transcription activator and transcription repressor. A direct activated target of PLZF is REDD1. REDD1 mediates PLZF-dependent down-regulation of TORC1 and it is responsible for the maintenance of spermatogonial progenitor cells in culture by mediating effective signaling from GDNF, while it is normally blocked by TORC1 activity. It has been postulated that the effect of REDD1 on TORC1 could also raise the possibility that REDD1 controls cell growth, tumorigenicity and senescence (36). 

PLZF activates PTEN/AKT/FOXO3 signaling pathways which can suppress prostate tumorigenesis (37). Deficiency of PLZF expression in prostate cancer is associated with tumor aggressiveness and metastasis (38). Shen et al. (39) showed that PLZF expression inhibited proliferation and metastasis via regulation of the interferon-induced protein with tetratricopeptide repeat 2 and increasing STAT1 protein level.

## Conclusion

Our data demonstrated that PLZF is expressed in unipotent Oct4+/VASA-SCCs in the basal compartment of adult testis seminiferous tubules. Our findings indicate that in comparison with unipotent SSCs, PLZF expression is not detectable in pluripotent ES-like cells which are directly derived from SCCs. Furthermore pluripotent ES cells do not express PLZF. Therefore, it could be proposed that PLZF represses and activates target genes which are specifically important for the maintenance of SSC. In the future, it would be interesting to anlyse the mechanism of PLZF down-regulation while SSCs shift to plutipotency and vice versa, during differentiation of pluripotent stem cells towards SSC *in vitro*. 
